# The Role of Immunotherapy in Non-transplant Eligible Multiple Myeloma

**DOI:** 10.3389/fonc.2020.00676

**Published:** 2020-05-06

**Authors:** Arthur Bobin, Hélène Gardeney, Florence Sabirou, Cécile Gruchet, Anthony Lévy, Laly Nsiala, Laura Cailly, Cécile Tomowiak, Jose Torregrosa, Stéphanie Guidez, Xavier Leleu

**Affiliations:** ^1^Service d'Hématologie et Thérapie cellulaire, Centre Hospitalier Universitaire de Poitiers, Poitiers, France; ^2^Unité Inserm CIC 1402, Poitiers, France; ^3^Université de Médecine et Pharmacie, Poitiers, France

**Keywords:** multiple myeloma, immunotherapy, non-transplant eligible, elderly, novel therapies

## Abstract

As the global population is aging and survival in multiple myeloma (MM) is increasing, treating older MM patients, redefined as non-transplant eligible (NTE), is becoming more frequent. Yet, treating these patients remains a real challenge especially because of a marked heterogeneity in the population and an increased susceptibility to treatment toxicity. Indeed, the balance between efficacy and safety must be considered at all time throughout the treatment history for these patients. Therefore, younger and older patients were historically treated in a very different way, even though the safety profile of most anti-myeloma drugs has drastically improved over the years. The emergence of immunotherapy (IT) has largely widened the therapeutic options available in MM and above all has allowed a therapy at optimal dose, and therefore optimal activity, for all patients independently of their frailty features, with no increase in safety issues. Among the novel anti-myeloma IT-based agents, anti-CD38 monoclonal antibodies (mAbs) are now becoming the new backbone of treatment for NTE patients, in association with lenalidomide and dexamethasone. Moreover, several new IT-based drugs are currently being developed and investigated either alone or in association; such as new anti-CD38 mAbs, anti-CD38 mAbs with many different combinations, but also the CAR-T cells, bispecific T-cell engager (BiTEs), or antibody drug conjugate (ADC) targeting BCMA. One would expect that immunotherapy will ultimately change and even transform the MM landscape, even for elderly patients. Immunotherapy represents a shift in treatment paradigm in MM as it provides truly efficient drugs with a very favorable safety profile.

## Introduction

The median age at diagnosis of multiple myeloma (MM), a plasma cell malignancy that typically occurs among elderly patients, is ~70 years old with nearly one third of the patients above 75 years old of age. Besides, the incidence of MM is expected to increase with the population aging, in parallel to the old and frail population. Also, while the patients with MM will surely live longer, the myeloma physicians will treat older and possibly more frail patients over time in the disease course. The overall issue with elderly MM patients is related to the impossibility for most of them to undergo an intensive treatment like autologous stem cell transplant (ASCT), with little prospect of debulking effectively the bone marrow with chemotherapy, and also few possibilities to harass the bone microenvironment in the tumoral niche.

The elderly MM population is very heterogeneous in real-life notably with great differences of age and physical decline features. Interestingly, while the MM features and characteristics at diagnosis are similar across older and younger adults, the inherent characteristics of the patients are truly distinct between both subsets. Thus, finding the optimal treatment approach for patients considered “elderly,” now logically renamed “non-transplant eligible” (NTE), has remained a challenge due to the marked heterogeneity of this population. Ultimately, advanced age is predictive of an increased risk of treatment-related toxicity (1), and thus monitoring and tailoring the drug management has become increasingly important for the safety of the patients.

Younger and older patients are usually treated in a very specific and different way, but the safety profile of most novel anti-myeloma drugs and families has drastically improved over the years so that the elderly are now eligible to innovative treatments, although not to intensification in most cases. Indeed, the emergence and development of immunotherapy (IT) has profoundly transformed the treatment landscape of cancer, particularly in MM, and thereby widen the treatment possibilities and enabled tailored strategies particularly for older patients, with possibly fewer adverse events. Novel agents were usually reserved for salvage or relapse therapy in the elderly, but with high efficacy and good tolerability they could find their way upfront and become the new treatment paradigm for this population.

## NTE Myeloma Patients is a Heterogeneous Patient Population With Very Different Objectives and Outcomes

The treatment selection for elderly patients with MM has historically been based upon chronological age and performance status, the frail patients having much less options. Over the years, it has become obvious that the NTE myeloma population is highly heterogeneous, and these 2 criteria are not sufficient to describe and categorize the entire population anymore. A careful patient assessment is essential before choosing the better treatment option and additional tools are therefore necessary to help physicians decide therapy apart from their clinical judgment alone.

The geriatric assessment (GA), made by an onco-geriatrician or directly by a hematologist with the help of specific geriatric scores, is now fully part of the guidelines for NTE MM, and therefore brought a major change to the definition of frailty (2). The International Myeloma Working Group frailty score (IMWG, range 0–5) (3), or similar scores such as the Revised Myeloma Comorbidity index (R-MCI, range 0–9, adding evaluation of renal and lung function) (4), have proposed a scoring system based on age, comorbidities, cognitive, and physical conditions allowing the identification of 3 subgroups of elderly patients: fit patients (IMWG score = 0, R-MCI ≤ 3), intermediate-fit (IMWG score=1, R-MCI 4–6) or frail (IMWG score ≥ 2, R-MCI >6). These frailty index scores predict mortality and the risk of toxicity, and were validated in several clinical trials (3–5). Other scores are under validation such as the UK Myeloma Research Alliance Risk Profile (MRP) (6) or the Hematology Oncology Frailty (HOF) scores (7).

By moving toward a personalized medicine in older MM patients and dividing them into several subgroups, treatment objectives might truly differ. While striving for the best possible response and the longer survivals (PFS and OS) for the first group of fit NTE patients, we should rather seek the right balance between efficacy and toxicity for the other 2 groups, namely intermediate fit and frail MM. Indeed, in the latter 2 groups, the need for safety control often negatively impact the need for efficacy, that is why the event-free survival (EFS), where the event includes a major treatment toxicity leading to discontinuation or relapse or death, appears as a more relevant endpoint. Reaching for longer EFS very nicely reflects the need for frail patients not to simply aim for survival with optimal tumor bulk control, but also reflects the need to extend survival by considerably improving the safety profile of the treatments. Nevertheless, in the next couple of years the advent of IT in older patients, given its excellent safety profile while keeping a profound activity on the tumor bulk, should finally reconcile the objectives of treatments for NTE myeloma across all the subgroups.

Currently, fit patients may be considered for upfront standard of care with triplet-based induction, and even possibly followed by ASCT, in a comparable approach to younger individuals (2). The objective tends to be identical to the patients that are transplant eligible, which is to achieve a deep response and therefore prolong survival. Intermediate-fit patients could also be treated with triplet-based induction approaches (2, 8), but not followed by ASCT, while being very cautious with drug management in order to avoid dose reductions that could impact the response. Finally, the frail population to whom safety matters even more because of the strong impact on survival should receive reduced-dose regimens, where doublet-based regimens play a major role, along with supportive care.

## An Increased Susceptibility to Treatment Toxicity

Patients with NTE myeloma, independently of their frail status, are more likely to be exposed to toxicity than younger patients, especially because of a decrease of their physiological reserve. Dealing with NTE myeloma requires to focus on matters beyond the only disease control: among the most frequent comorbidities are renal dysfunction, peripheral polyneuropathy (PN), cardiovascular diseases, diabetes, venous thromboembolism or an impaired nutritional status, not speaking of loss of memory, and any type of cognitive impairments (9, 10). A Danish study on 2,190 MM patients highlighted that the MM population had increased comorbidity compared to the control (OR 1.4, 1.1–1.7), and patients with registered comorbidity had increased mortality compared with patients without comorbidity (HR 1.6, 1.5–1.8) (11). In a meta-analysis of 1,435 patients treated with thalidomide and/or bortezomib it was detected that age and organ damage was associated with reduced overall survival (12). The risk of death was more important in patients with renal failure (HR 2.02, 95%CI: 1.51–2.70; *p* < 0.001), in those who experienced grade 3–4 infections, cardiac or gastrointestinal adverse events during treatment (HR 2.53, 95%CI: 1.75–3.64; *p* < 0.001) and in those who required drug discontinuation due to adverse events (HR 1.67, 95%CI: 1.12–2.51; *p* = 0.01).

Therefore, the choice of upfront therapy for NTE MM must take into account treatment-related toxicity, pre-existing comorbidities, polypharmacy and the alteration of quality of life it may implies. Given the numerous options available at hand these days, particularly with immunomodulatory (IMiDs) drugs, proteasome inhibitor (PI), alkylating agents, and corticosteroids, and recently immunotherapy (IT) the main challenge is to find the appropriate regimen in order to reduce side effects that could jeopardize the clinical benefits.

## Current Standard of Cares for the Elderly MM Patients

Treatment options for NTE patients greatly evolved over the past decade. Overall, the therapeutic strategy moved from melphalan-based induction regimens to lenalidomide-based associations, which is now the backbone of most treatment for NTE patients. In fact, there is still one last melphalan-based combination with IT (daratumumab-MPV) which probably will last until IT with Rd will be available worldwide.

Although the MPV regimen was one of the best standard of care for NTE patients, and available in most countries worldwide, it was not so well-tolerated and could hardly be given to patients for more than 12 cycles in real life given the neurological toxicity of bortezomib and the risk of myelodysplasia with melphalan. Indeed, in the VISTA trial (MPV vs. MP) 46% of patients had grade 3–4 toxicity and 15% had to discontinue the treatment due to adverse events (AE), despite a 3-year OS rate of 68.5% (13). In that context, the new standard of care lenalidomide and dexamethasone (Rd) was appealing, improving the safety signature but also the convenience with an easy entirely oral administration. However, studies of Rd have reported up to 45% serious AEs for the patients receiving lenalidomide, with dose modifications applied to 69% of lenalidomide patients (14), and the control of MM could be also be improved particularly among high risk MM.

Therefore, the addition of bortezomib to Rd was the logical next step to improve the activity of bortezomib and lenalidomide altogether for NTE myeloma upfront. The phase III SWOG S0777 trial compared VRD vs. Rd and the rates of overall response were better in the VRd group vs. Rd (82 vs. 72%) as long as the rate of complete response or better (≥CR) (15.7 vs. 8.4%) (8). This lenalidomide-based triplet regimen had also increased the toxicity signature in a certain degree, the grade ≥3 AEs rate increased with VRd compared to Rd (82 vs. 75%). Consequently, frail patients are often only treated with doublet-based regimens, certainly safer but also less active overall (8).

Another important aspect of the treatment of myeloma comes to the optimal duration of these treatments. The objective of continuous therapy is to prolong or improve the depth of response by further controlling the tumor mass and the bone marrow microenvironment, and ultimately to allow immune reconstitution, in order to extend PFS. It was shown that continuous treatment could be a better way to improve the treatment benefit for NTE patients given the current drugs available for now on. This clearly is a benefit provided by Rd regimen over MPV for example. Yet, the benefit of the continuous treatment on OS has never been demonstrated (14, 15).

## Dexamethasone and the Risk to Increase Toxicity in NTE Myeloma

The long-time use of corticosteroids in MM is being challenged in the modern era. The IFM (“Intergroupe Francophone du Myélome”) in the old time, and more recently in 2010 the ECOG E4A03 study (16), demonstrated the toxicity of dexamethasone particularly in NTE patients. It was shown that lenalidomide and low-dose dexamethasone was associated with lower toxicity (≥grade 3 AE, 52 vs. 35%) without losing in efficacy because short-term OS was also better (96 vs. 86%). The practice has then evolved toward decreasing the dose of dexamethasone to once weekly instead of blocks of 4 days in a row.

Even more recently, the randomized phase III RV-MM-PI-0752 study compared standard continuous Rd vs. Rd induction and R maintenance in elderly intermediate-fit patients (17), investigating the balance between efficacy and safety in elderly intermediate-fit patients. The preliminary results, yet to be published, demonstrated improved median-EFS in the Rd-R group compared to the Rd continuous dexamethasone group, respectively, 9.3 and 6.6 months, with a median follow up of 25 months. R discontinuation and dose reductions for AEs were lower in the Rd-R group in line with the permanent interruption of dexamethasone. This study demonstrates that the final dose intensity of R monotherapy is better when dexamethasone is interrupted and therefore the toxicity profile of dexamethasone decreased. Sparing steroids in the lenalidomide treatment for the frail population is therefore feasible without a negative impact on survival. One can therefore anticipate that in the future dexamethasone is going to be rapidly tapped down or even permanently discontinued much earlier in the treatment phase in the NTE population. Although, this could mean that lenalidomide will end up being given to patients as an equivalent of monotherapy, more like a maintenance treatment than an optimal debulking regimen.

Given that corticosteroids can be poorly tolerated for NTE patients causing mood swings, insomnia, diabetes or high blood pressure, the idea would be to stop dexamethasone at some point of the treatment to favor compliance with the other drugs or simply to dispense patients from dexamethasone in the first place, without losing in efficacy. Consequently, how to optimize MM treatments in general, and lenalidomide-based regimen specifically, in association to other drugs but sparing patients from dexamethasone? One option would be to combine bortezomib to lenalidomide (VR, without dex); although the neurotoxicity of bortezomib will remain an issue for the patients. In that context, the combination of lenalidomide to immunotherapy (IT) is more appealing, and might create a new backbone in order to spare the use of corticosteroids while maintaining a high efficacy. Several studies are investigating a combination of drugs to IT so that it could possibly spare patients from dexamethasone. Indeed, the phase II Ixa-dara trial (NCT03757221) is studying the combination of ixazomib and daratumumab, without dexamethasone, in elderly RRMM and the phase III IFM-2017-03 (NCT03993912) is trying subcutaneous (SC) daratumumab plus lenalidomide vs. Rd alone for frail NTE NDMM.

## Immunotherapy, the Hype of Myeloma Treatment Paradigm

Immunotherapy is certainly one of the most impressive and successful drug development in multiple myeloma, with the anti-CD38 monoclonal antibodies (mAbs) on top of the class. Actually, the anti-CD38 mAbs drug family has already profoundly transformed the treatment landscape of myeloma, including in upfront therapy. Besides, immunotherapy also led to the advent of new MM target and the discovery of novel class drugs like anti-SLAMF7 or checkpoint inhibitors. Although anti-CD38 first-in-class daratumumab was not the first IT developed in MM, it surely is the most impressive these days by far in relation to novel IT-based mechanisms of action.

### Daratumumab

Daratumumab is the first fully humanized mAb targeting CD38 and is already approved for relapsed/refractory MM (RRMM) combined with either bortezomib (18) or lenalidomide (19) in early relapsed MM or in monotherapy (20) in late relapse MM.

In the 4-year update analysis of CASTOR trial (21) (dara-Vd vs. Vd) the median PFS was 16.1 months in the daratumumab group and was 7.1 months in the control group (HR, 0.31; 95% CI, 0.25–0.39), after a median follow-up of 47 months. With this longer follow up the discontinuation rates due to treatment emergent AEs were similar for D-Vd vs. Vd (10 vs. 9%). In the initial publication (18) we could see that dara-Vd advantage was also seen across the ≥65 years old groups (HR 0.35, 95% CI 0.22 to 0.53). We could count 96 patients (38.2%) between 65 and 74 years old and 23 (9.2%) ≥75 years old, but no data are available for frail patients. In the recent update of the POLLUX trial (22) (dara-Rd or Rd) the median PFS was significantly higher with dara-Rd vs. Rd, respectively, 45.8 vs. 17.5 months with a median follow up of 51.3 months (HR, 0.43; 95% CI, 0.35–0.54), and 43.4% of the patients were 65–74 years old and 10.1% ≥75 years old. As in the CASTOR trial, the treatment discontinuation was similar in both arms (D-Rd 16 vs. 15% Rd). Interestingly, as seen in the first publication (19), the patients who benefited the most from the association dara-Rd were the ≥75 yo group (HR 0.11, 95% CI 0.02–0.51).

Daratumumab was also studied in association with new IMId pomalidomide in the APOLLO trial for RRMM (23). At a median follow up of 13.1 months, the median PFS of dara-poma-dex was 8.8 months (95% CI, 4.6–15.4) and median OS was 17.5 (95% CI, 13.3-NE) months. The ORR was 60% and among patient with ≥CR, 29% were MRD negative at a threshold of 10^−5^. Of note, 42% of the patients were 65– <75 years old and 8% >75 years old. Except the higher incidence of neutropenia (without an increase in infection rate) and infusion related reaction, the safety profile was similar to that of the control arm pomalidomide and dexamethasone.

Moreover, a matching-adjusted indirect comparison (MAIC) demonstrated that dara-VTD (as in the CASSIOPEIA trial) was superior to VRD in transplant-eligible patients. This result confirms and underlines the efficacy of the addition of anti-CD38 mAb in comparison to conventional MM regimen (24).

### Anti CD38 mAb for NTE Patients

Based on the results of these previous studies and the apparently excellent tolerability, daratumumab-based trials were specifically designed for NDMM ineligible for ASCT. Phase III ALCYONE trial (25) analyzed the combination dara-MPV vs. MPV for a fixed duration of 9 cycles induction in both groups, with daratumumab monotherapy continued as maintenance in the Dara-MPV arm. Median age was 71 years old in the 2 groups. The 18-month PFS rate was 71.6% in the daratumumab group and 50.2% in the control group (HR 0.5, 95% CI 0.38–0.65). Furthermore, in the daratumumab group 23.3% of the patients had MRD negative as compared with 6.2% in the control group (*p* < 0.001). This data is not yet available according to frailty features of the patients. Serious AE were higher in the daratumumab group (41.6 vs. 32.5%), but the rate of discontinuation was lower with dara-VMP (4.9 vs. 9%). The results provided by the updated analysis were consistent with the initial results, the median PFS was higher for patients in the daratumumab group (36.4 vs. 19.3 months) with a median follow up of 40.1 months, and the response rates seemed to deepen over time with the addition of daratumuab (26). Besides, there were no new safety concerns with this longer-term follow-up. Similarly, daratumumab was also investigated with lenalidomide and dexamethasone upfront for transplant ineligible in the phase III MAIA trial (27), D-Rd or Rd until progression. The primary analysis of MAIA has shown that the estimated 30-month PFS was 70.6% in the daratumumab group and 55% in the control group (HR 0.56, 95% CI 0.43 to 0.73). In addition, 24.2% of the patients had MRD 10^−5^ negative rate. A benefit could also be seen for ≥75 years old patients with a hazard ratio of 0.63 (CI95% 0.44-0.92). Serious AEs were similar between the 2 groups (DRd 62.9 vs. 62.7% Rd). With a 9-months longer follow up (28), the benefit of DRd could still be seen, the estimated 36-month PFS rate was 68 vs. 46% (DRd vs. Rd). Also, less patients discontinued the treatment due do treatment related AE in the daratumumab arm (9 vs. 18%).

### Future Daratumumab-Based Associations for NTE

The phase 3 CEPHEUS trial will evaluate the safety and efficacy of dara-VRd vs. VRd as initial therapy in this NTE patient population (NCT03652064). Daratumumab will also be associated with ixazomib, lenalidomide, and dexamethasone as a quadruplet regimen in a phase II trial (NCT04009109). Eventually, daratumumab will also still be part of the treatment of elderly RRMM as in the Idara trial (ixazomib-daratumumab no dexamethasone) (NCT03757221).

### Isatuximab

Isatuximab is a new anti-CD38 chimeric monoclonal antibody for therapeutic use under development that does not exactly show identical effect in MM cells because of different mechanisms. The association of isatuximab, pomalidomide and dexamethasone was tested in the ICARIA trial for RRMM and showed a longer PFS vs. pomalidomide and dexamethasone alone (11.5 vs. 6.5 months) (29). Median age of the population was 68 years old and the oldest patient was 74 years old, so it is quite safe to say that this combination could also benefit NTE patients. Still, we will have to wait for the results of the phase III trial IKEMA for RRMM where isatuximab will be associated to novel PI carfilzomib. Isatuximab will also be specifically tested upfront for NTE patients in the phase III IMROZ study (NCT03275285), isatux-VRd vs. VRd (NCT03319667). In the experimental arm, isatux-VRD will be administered for 4 cycles followed by continuous treatment with isatux-Rd.

### Safety Profile and QOL of Anti-CD38 mAbs

The most frequent adverse events for anti-CD38 mAbs when used in monotherapy are infusion-related reactions (IRR) during the first injections. Overall, the safety profile of anti CD38 mAbs appears to be very manageable in NTE patients including the frail ones. Meanwhile, it should probably be noted that the infection rate of any grade was higher in the DRd group of MAIA, 86.3 vs. 73.6%, and particularly pneumonia (22.5 vs. 12.6%). These results were consistent to those from ALCYONE where indeed the addition of daratumumab to MPV also resulted in more infections (any grade infection dara-MPV 66.8 vs. 48% MPV). Part of the explanation probably lies in the worsening of the hypogammaglobulinemia due to the targeting of non-clonal plasma cells by anti-CD38 mAbs. Nevertheless, it has to be noted that in these trials the exposure to the experimental treatment was slightly longer than in the control group (ALCYONE, median duration of treatment: D-VMP 63.9 vs. 52.1 weeks VMP; MAIA: DRd 25.3 vs. 21.3 months Rd), so that it could supposedly have influenced the infection rate. The proportion of old and very old patients was notable in both trials, 29.7 and 43.5% patients ≥75 years old for ALCYONE and MAIA, respectively, but similar in studied and control arms, and likely not an explanation herein.

Of note, the administration of daratumumab will change from intravenous (IV) to subcutaneous (SC) with a flat dose of 1800 mg, providing a major benefit for the patients particularly concerning their quality of life. The phase III COLUMBA study (30) (dara-IV vs. dara SC for RRMM) showed that dara-SC was not inferior to dara-IV with comparable safety profile. Although not specifically designed for NTE patient, we could imagine that this subgroup could really take advantage to this new form of administration.

### Anti-SLAMF7 Monoclonal Antibodies

Plasma cells express the antigen SLAMF7 directly targeted by the humanized immunoglobulin mAb elotuzumab. It has been demonstrated that elotuzumab induced cell death using antibody-dependent cell mediated cytotoxicity (ADCC). Interestingly, while elotuzumab has a killing effect on tumor cells, it might have an activating effect on certain effector cells of interest that are involved in the ADCC mechanism of action of elotuzumab. Elotuzumab was tested in several combinations for myeloma, including older patients such as elotuzumab with lenalidomide and dexamethasone (elo-Rd) (31) or elotuzumab with bortezomib and dexamethasone (elo-Vd) (32). In the phase III ELOQUENT-2 trial (31) that evaluated elotuzumab-Rd vs. Rd, 20% of patients were ≥75 years. Median PFS in the elotuzumab group was 19.4 vs. 14.9 months in the control group (HR 0.70, 95% CI 0.57–0.85). The benefit was persistent in the subgroup analysis for patient aged 65 or older with a hazard ratio of 0.65 (95% CI 0.5–0.85). Most recently, the final OS analysis was presented at the 17th IMW workshop (Dimopoulos *et al*. abstract OAB-021) with a follow up of 71 months, and the median OS was 48.3 months for Elo-Rd vs. 39.6 months with Rd. The safety analysis was consistent with the primary analysis, grade 3–4 serious AEs (ERd vs. Rd) 53 vs. 40%). Elotuzumab was also investigated in association with bortezomib in a phase II study, Elo-Vd vs. Vd, for RRMM (32). Patients aged 75 or older represented 19% of the whole population. The combination Elo-Vd showed a trend for an improved PFS, median PFS was 9.6 months with Elo-Vd vs. 6.9 months with Vd (HR 0.72, 95% CI 0.49–1.06). This trend could also be seen for patients ≥65 (HR 0.71, 0.43–1.17). Elotuzumab is also currently being evaluated with carfilzomib, lenalidomide and dexamethasone (Elo-KRd) for previously untreated NDMM for both transplant and non-transplant eligible patients (NCT02969837).

Elotuzumab in combination with IMiDs seems more appealing and clinical trials focusing on this association are ongoing. Interestingly Elo-Rd was well-tolerated in a population with renal impairment (33).

### Checkpoint Inhibiton

Checkpoint inhibitors were developed in many cancer and showed great activity, so it was only a matter of time before it was explored in MM. Overall, despite the development of several investigational drugs and their combinations with other anti-MM drugs, there is no obvious demonstration that this IT could bring another viable option to the field of MM (34, 35). Plus, its feasibility in elderly myeloma patients has never been explored.

## What Will the Future Look Like?

A better knowledge of multiple myeloma pathophysiological mechanisms already brought us innovative drugs such as novel PI, IMiDs or monoclonal antibodies, but this is just the beginning of what could truly be a new era for MM patients and clinicians. Indeed, the development of armed IT, such as the CAR-T cell therapy, the T cell engagers or antibody-drug conjugates, is growing fast and will allow us to provide the MM community with novel therapeutic approach, probably for both transplant eligible (TE) and NTE patients. The concept of “living drugs” will not be restricted to the young patients.

### Chimeric Antigen Receptor T Cell (CAR-T)

CAR-T cell therapy is one of the most recent immunologic approaches and made a drastic change in the hematological field. CAR-T are genetically modified autologous T-cells expressing a specific receptor to a tumor antigen that causes T-cell activation after binding. CAR-T cell therapy is rapidly gaining attention in MM principally using BCMA-specific CAR-T (36–40). Information focusing on older patients is sparse at the moment and we do not exactly know if it could benefit this group of population. In the published phase I study using bb2121, an anti-BCMA CAR-T cell, patients up to 75 years old received the treatment. Among the 33 heavily pre-treated patients (median number of previous regimens = 7) the objective response rate was 85% (95% CI, 68.1–94.9), with 45% ≥ CR. No subgroup analysis focusing on the older patients is available.

### Bispecific Antibodies

Immunotherapy has also led to the development of bispecific antibodies (BsAbs) which have 2 antigen-recognition domains. Bispecific T-cell engagers (BiTEs) are a sub-type of BsAbs that allow T-cell proximity to tumor cells generating T-cell proliferation and leading to tumor cell lysis. Several targets for BiTEs in MM are under investigation such as BCMA, CD38, or CD138 (41, 42). The results presented at the 2019 ASCO congress from the phase I dose-escalation study with AMG 420 (anti BCMA BiTE) concerned 42 patients having RRMM with a median age of 65 years. The oldest patient was 79 years old. Thirteen patients responded to treatments (31%) but 45% experienced serious AEs. Data concerning elderly patients will be expected to further determine if BiTEs can be an additional option for them.

### Antibody **Drug Conjugate** (ADC)

Apart from BiTEs, antibody-drug conjugate (ADC) are also currently being developed. Belantamab-mafodotin (GSK2857916) is an anti-BCMA MAb conjugated to auristatin F through a non-cleavable linker that was explored in the phase I DREAMM-1, whose results (60% ≥ PR, including 2 sCR and 3 CR; median PFS was 12 months) led to the DREAMM-2 trial (phase II) (43). Belantamab-mafodotin demonstrated clinically substantial overall response rate (ORR) for RRMM in the DREAM-2 trial. Several other studies are planned for ADC's development and especially the DREAMM-9 study that will only concern ineligible to transplant patients. In this future phase III study the patients will receive belantamab-mafodotin plus VRd or VRd alone.

These new approaches with IT are offering multiple different possibilities to treat patients suffering from MM, and although the data are lacking concerning NTE and frail patients, one can expect that they will also benefit from this progress. If as expected this new armamentarium results in deep responses the previously listed premises across this review could change drastically. Indeed, as long as treatment toxicity remains modest, reaching for MRD negativity even for older patients will be the ultimate goal. Therefore, the optimal duration of treatment is questioned; MRD-negative patients could stop maintenance or continuous treatment while also avoiding long-term toxicities. MRD-adaptative treatment is massively being experienced in clinical trials at the moment. Deepening response rate could finally put an end to prolonged therapy, which remains psychologically, and physically difficult to keep up with for the patients. Furthermore, in addition to being less cytotoxic novel agents could also lead to immune reconstitution a most certainly critical objective in MM.

## Conclusion

For a long time, clinical trials specifically designed for elderly patient, or not-transplant eligible, were lacking. This subgroup, the most represented subgroup of patients among MM, has progressively gained more interest and therefore the therapeutic strategy has evolved. The development of more suitable drugs to NTE MM patients has definitely helped the field to better treat those patients. However, the past 20 years have taught the MM physicians a lot regarding the better treatment approach for NTE frail MM patients, the limitations of the drugs at hand, and the need to further optimize control of the disease with a balance safety/efficacy. Depth of response, duration of treatment and safety control of prolonged therapy are currently the major objectives, but for some subgroups of patients particularly the frail more an issue than a goal. In that regard, a new drug family was needed, and it seems that immunotherapy could solve many of the problems listed above, particularly anti-CD38 monoclonal antibodies, but more is to come.

Following the results of the MAIA trial, the combination of anti-CD38, lenalidomide and dexamethasone will probably become the new most effective and among the safest standard of care for NTE MM, introducing immunotherapy the best way in MM (Table 1). The future will likely see further developments of immunotherapy in MM, with the objective to solve the most important question of the next 5 to 10 years, will MRD negativity lead to permanent treatment discontinuation?

**Table 1:** Evolution of treatment standards for non-transplant eligible patients.

	**MPV^13^**	**Rd^15^**	**VRd^8^**	**Dara-MPV^25^**	**DRd^27^**
ORR, %	71	81	81.5	90.9	92.9
≥CR, %	30	22	15.7	42.6	47.6
MRD 10^−5^, %	-	-	-	22.3	24.2
Median-PFS, months	20.5	26	43	Not reached	Not reached
Estimated PFS, %	-	-	-	18 months: 71.6%	30 months: 70.6 %
Median-OS, months	61	59.1	75	Not reached	Not reached
Serious AE, %	91	-	-	41.6	62.9
Treatment discontinuation, %	15	-	-	4.9	7.1
Subgroups of interest.					
High risk patients HR (95%IC)	-	1.27 (0.81–2.01)	-	0.78 (0.43–1.43)	0.85 (0.44–1.65)
≥75 years old HR (95%IC)	-	0.78 (0.60–0.99)	-	0.53 (0.32–0.85)	0.62 (0.44–0.92)
ECOG ≥2 HR (95%IC)	-	0.82 (0.10–1.11)	-	0.51 (0.29–0.89)	

## Author Contributions

AB and XL wrote the manuscript. AB, HG, FS, CG, AL, LN, LC, CT, JT, ST, and XL critically reviewed the manuscript.

### Conflict of Interest

The authors declare that the research was conducted in the absence of any commercial or financial relationships that could be construed as a potential conflict of interest. The handling Editor declared a past co-authorship with one of the authors XL.
